# Role of African Swine Fever Virus Proteins EP153R and EP402R in Reducing Viral Persistence in Blood and Virulence in Pigs Infected with BeninΔDP148R

**DOI:** 10.1128/JVI.01340-21

**Published:** 2022-01-12

**Authors:** Vlad Petrovan, Anusyah Rathakrishnan, Muneeb Islam, Lynnette C. Goatley, Katy Moffat, Pedro J. Sanchez-Cordon, Ana L. Reis, Linda K. Dixon

**Affiliations:** a The Pirbright Institute, Pirbright, Woking, Surrey, UK; University of North Carolina at Chapel Hill

**Keywords:** African swine fever virus, EP153R, EP402R, macrophages, persistence, virulence determinants

## Abstract

The limited knowledge on the role of many of the approximately 170 proteins encoded by African swine fever virus restricts progress toward vaccine development. Previously, the DP148R gene was deleted from the genome of genotype I virulent Benin 97/1 isolate. This virus, BeninΔDP148R, induced transient moderate clinical signs after immunization and high levels of protection against challenge. However, the BeninΔDP148R virus and genome persisted in blood over a prolonged period. In the current study, deletion of either EP402R or EP153R genes individually or in combination from BeninΔDP148R genome was shown not to reduce virus replication in macrophages *in vitro*. However, deletion of EP402R dramatically reduced the period of infectious virus persistence in blood in immunized pigs from 28 to 14 days and virus genome from 59 to 14 days while maintaining high levels of protection against challenge. The additional deletion of EP153R (BeninΔDP148RΔEP153RΔEP402R) further attenuated the virus, and no viremia or clinical signs were observed postimmunization. This was associated with decreased protection and detection of moderate levels of challenge virus in blood. Interestingly, the deletion of EP153R alone from BeninΔDP148R did not result in further virus attenuation and did not reduce the period of virus persistence in blood. These results show that EP402R and EP153R have a synergistic role in reducing clinical signs and levels of virus in blood.

**IMPORTANCE** African swine fever virus (ASFV) causes a disease of domestic pigs and wild boar which results in death of almost all infected animals. The disease has a high economic impact, and no vaccine is available. We investigated the role of two ASFV proteins, called EP402R and EP153R, in determining the levels and length of time virus persists in blood from infected pigs. EP402R causes ASFV particles and infected cells to bind to red blood cells. Deletion of the EP402R gene dramatically reduced virus persistence in blood but did not reduce the level of virus. Deletion of the EP153R gene alone did not reduce the period or level of virus persistence in blood. However, deleting both EP153R and EP402R resulted in undetectable levels of virus in blood and no clinical signs showing that the proteins act synergistically. Importantly, the infected pigs were protected following infection with the wild-type virus that kills pigs.

## INTRODUCTION

African swine fever virus (ASFV) causes an acute hemorrhagic fever in domestic pigs and wild boar with case fatality approaching 100%. In contrast, its wildlife hosts in Africa, warthogs, bushpigs, and soft ticks of *Ornithodoros* species that inhabit warthog burrows, can be persistently infected but show few disease signs (1, 2). ASFV has a very high economic impact in affected countries, which now include most of sub-Saharan Africa, parts of Russia, Eastern Europe, and 11 European Union countries. The spread to China in 2018 and subsequent spread to Southeast Asia resulted in death or culling of more than 7 million pigs and a decrease of the Chinese herd by about 40% (FAO Situation Update, OIE WAHIS; https://www.fao.org/ag/againfo/programmes/en/empres/ASF/situation_update.html). Since there are no vaccines or targeted therapeutics currently available, control relies on implementing strict biosecurity measures and culling of infected herds.

ASFV is a large DNA virus with a linear double-stranded genome varying in size from 170 to more than 190 kbp. The virus is the only member of the *Asfarviridae* family and has a predominantly cytoplasmic replication. The virus genome contains up to 167 genes, including many that are not required for the virus to replicate in cells but have roles in interactions with the host to facilitate its survival and transmission (3). For example, the virus codes for several proteins that help the virus to evade the host innate immune response, such as the type I interferon (IFN) response and apoptosis (4). Deletion of genes that inhibit type I interferon (IFN) response, including members of the multigene families (MGF) 360 and 505 and DP96R (also designated UK) (5–10), can reduce the virulence of the virus in pigs and induce an immune response to protect the animal against lethal challenge with a related virulent virus.

ASFV also codes for two transmembrane glycoproteins that are not essential for virus replication in cells, pEP402R (CD2v) and pEP153R (11, 12). The EP402R gene codes for a type I transmembrane protein with similarity in its extracellular domain to the host CD2 protein. This virus protein pEP402R, also designated CD2v or CD2-like, is required for the binding of red blood cells to infected macrophages (hemadsorption [HAD]) (13, 14). It is also presumed to cause binding of red blood cells to extracellular virions, as 95% of virus in blood from infected pigs was shown to be in the red blood cell fraction (15). The CD2v protein was also suggested to have a role in the ASFV-induced inhibition of *in vitro* proliferation of lymphocytes in response to mitogens, since deletion of the gene abrogated this effect (16). Interactions of proteins, including SH3P7/mAbp1 and AP1, with the cytoplasmic tail were demonstrated, suggesting that these may be involved in intracellular trafficking of the protein through the Golgi apparatus (17, 18). The CD2v protein is the only virus protein to be detected on the surface of extracellular virions (19). Interestingly, pigs immunized with a recombinant CD2v expressed in baculovirus showed reduced viremia after challenge with E75 isolate (20). An involvement of cell-mediated protection was suggested since several T-cell epitopes were mapped using overlapping CD2v peptides (21).

The EP153R type II transmembrane protein contains a predicted C-type lectin domain. C-type lectins are Ca^2+^-dependent glycan-binding proteins that are involved in cell-cell adhesion playing key roles in both innate and adaptive immune responses. For example, C-type lectin receptors (CLRs) are important for recognition and capture of pathogens, as these pattern recognition receptors (PRRs) have a high affinity for their ligands, which results in internalization of the pathogens (22). The EP153R protein has been demonstrated to augment the HAD induced by the CD2v protein (23) and to have roles in inhibiting apoptosis mediated by the p53 pathway and in reducing the surface expression of swine leukocyte antigen I (SLAI) (24, 25).

Our previous experiments showed that deletion of the DP148R gene from the genome of the virulent Benin 97/1 genotype I isolate moderately attenuated the virus in pigs and induced high levels of protection against lethal challenge with parental virus (7). Genotype I is the predominant ASFV genotype circulating in Central and West Africa as well as parts of South Africa and in Sardinia in Europe. Studies on genotype I viruses are therefore important for eventual development of vaccines for Africa, and the information gained is likely to be applicable to other virus genotypes. For example, the genes studied here, EP402R and EP153R, are present in almost all virulent isolates. In pigs immunized with BeninΔDP148R, a peak of viremia was detected in blood at 5 or 6 days postimmunization coincident with clinical signs. After this, clinical signs were not observed but virus genome in blood declined slowly over a period of about 60 days. Infectious virus declined more rapidly and was not detected after about days 28 to 30 (7). In our current study, we investigated the hypothesis that virus binding to red blood cells, mediated by the CD2v protein, may prolong the persistence of virus in blood. Therefore, we deleted EP402R or EP153R genes either alone or in combination from the genome of the BeninΔDP148R virus and carried out immunization and challenge experiments in pigs. The results confirmed that deleting the EP402R gene reduced dramatically the period of infectious virus and genome persistence in blood. Transient clinical signs and a peak of viremia were still observed postimmunization of pigs, and high levels of protection were observed. Deletion of the EP153R gene from the BeninΔDP148RΔEP402R genome further attenuated the virus, and no clinical signs or viremia were observed. However, deletion of EP153R alone from BeninΔDP148R revealed a similar clinical outcome compared with BeninΔDP148R and a prolonged period of virus persistence in blood. Overall, using a genetic backbone of a previously attenuated virus, we provided new insights into the role of the CD2v and EP153R proteins in virus virulence and persistence in blood.

## RESULTS

### Generation of recombinant viruses.

**FIG 1:**
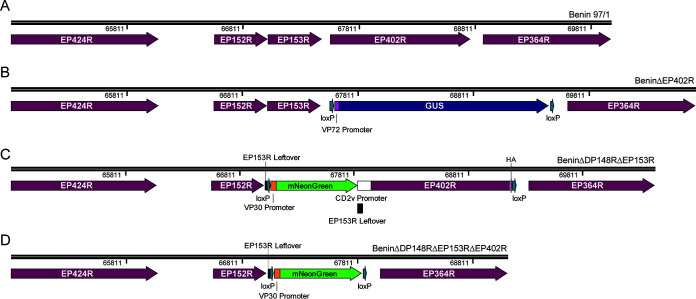
Schematic diagram depicting the deletion of EP153R and/or EP402R genes from genotype I ASFV. EP402R was deleted by homologous recombination between transfer plasmid pΔEP402R-VP72GUS and parental Benin 97/1 isolate (A), and the resultant, BeninΔEP402R (B), was purified using limiting dilutions. Using a sequential deletion method, DP148R was then further deleted to produce BeninΔDP148RΔEP402R via single-cell isolation, combined with limiting dilutions. With a previously described BeninΔDP148R, EP153R was deleted by homologous recombination to produce BeninΔDP148RΔEP153R (C), which contains mNeonGreen reporter marker. Likewise, using BeninΔDP148R as the parental virus, a triple gene-deleted virus, BeninΔDP148RΔEP153RΔEP402R (D), was produced. Both recombinant viruses were isolated and purified using FACS and limiting dilutions.

**BeninΔDP148RΔEP402R.** A two-step sequential deletion method was applied to produce the recombinant ASFV BeninΔDP148RΔEP402R (Fig. 1B). In the first step, EP402R (genome position: 67,567 to 68,775) was deleted from the virulent Benin 97/1 isolate (Fig. 1A) using homologous recombination. This virus, BeninΔEP402R, was subsequently cultured and used in the second step to construct recombinant ASFV BeninΔDP148RΔEP402R.

Using a single-cell isolation method for producing recombinant ASFV (26), the second step in the production of BeninΔDP148RΔEP402R involved the inclusion of a fluorescent reporter gene TagRFP-T in place of the deleted DP148R gene. Using fluorescence-activated cell sorter (FACS), single cells expressing the TagRFP-T reporter were isolated and the recombinant virus was purified with a combination of single-cell isolation and limiting dilutions.

**BeninΔDP148RΔEP153RΔEP402R and BeninΔDP148RΔEP153R.** A similar approach of single-infected-cell isolation and purification was used to generate recombinant ASFV BeninΔDP148RΔEP153R and BeninΔDP148RΔEP153RΔEP402R in which either EP153R (genome position: 67,051 to 67,491) alone was deleted from BeninΔDP148R or both genes EP153R and EP402R (genome position: 67,051 to 68,775) were deleted simultaneously. These gene(s) were replaced by mNeonGreen under the control of VP30 promoter in the attenuated BeninΔDP148R virus (7) (Fig. 1C and D). In both viruses, 21 bp at the 5′ end of EP153R was left in the recombinant viruses because this stretch of sequence may contain the termination signal for the adjacent EP152R gene (27). The expected deletions of genes in the recombinant virus were confirmed via PCR analysis and Sanger sequencing. The purified recombinant virus stock was propagated on porcine bone marrow cells (PBMs), and titrations were performed in quadruplicate on PBMs collected from different pigs.

### Growth curves.

**FIG 2:**
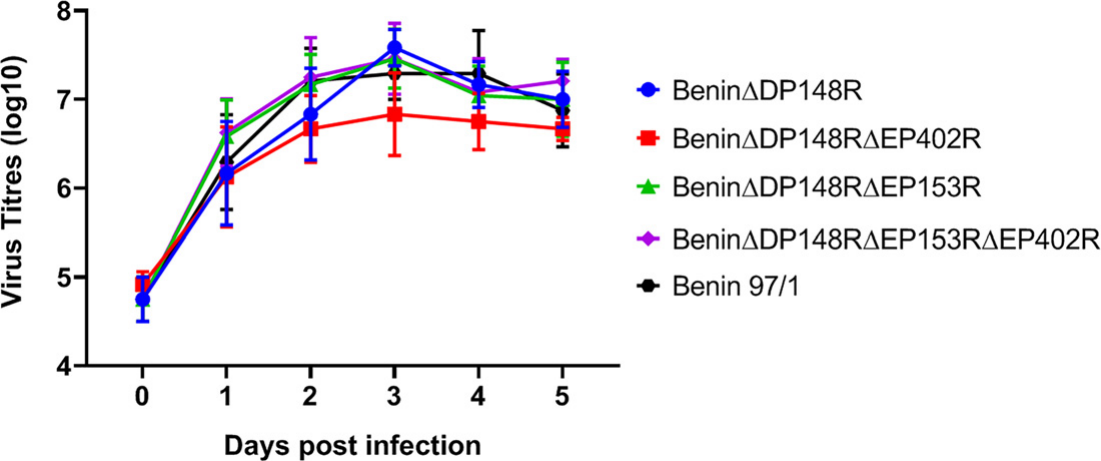
Replication of the recombinant gene-deleted ASFV viruses compared to the wild-type Benin 97/1 strain. Purified PBMs from 2 different pigs were infected with viruses at MOI 0.01 in triplicates. Viruses were harvested from both cells and supernatants at different time points and titrated on PBMs in quadruplicates. Virus titers are presented as log_10_ HAD_50_/mL for viruses BeninΔDP148R and Benin 97/1 or TCID_50_/mL for viruses BeninΔDP148RΔEP153R, BeninΔEP148RΔEP153R, and BeninΔDP148RΔEP153RΔEP402R.

Porcine bone marrow cells were infected with the Benin 97/1, BeninΔDP148R, BeninΔDP148RΔEP402R, BeninΔDP148RΔEP153RΔEP402R, and BeninΔDP148RΔEP153R at a multiplicity of infection (MOI) of 0.01 to determine if deletion of the genes affected the ability of the virus to replicate *in vitro*. At different days postinfection (1–5), total virus harvested from cells and supernatant was titrated. The results showed no significant difference between the kinetics and the levels of virus replication of the recombinant viruses and parental Benin 97/1 isolate (Fig. 2). Virus titers reached a plateau of approximately 10^7^ 50% tissue culture infective dose (TCID_50_)/mL between 24 and 48 h postinfection and were maintained for the remainder of the culture time. The results show that deletion of the DP148R, EP153R, EP402R, or EP153R and EP402R gene did not significantly alter the ability of the virus to replicate in culture.

### Clinical observations in immunized and challenged pigs.

**FIG 3:**
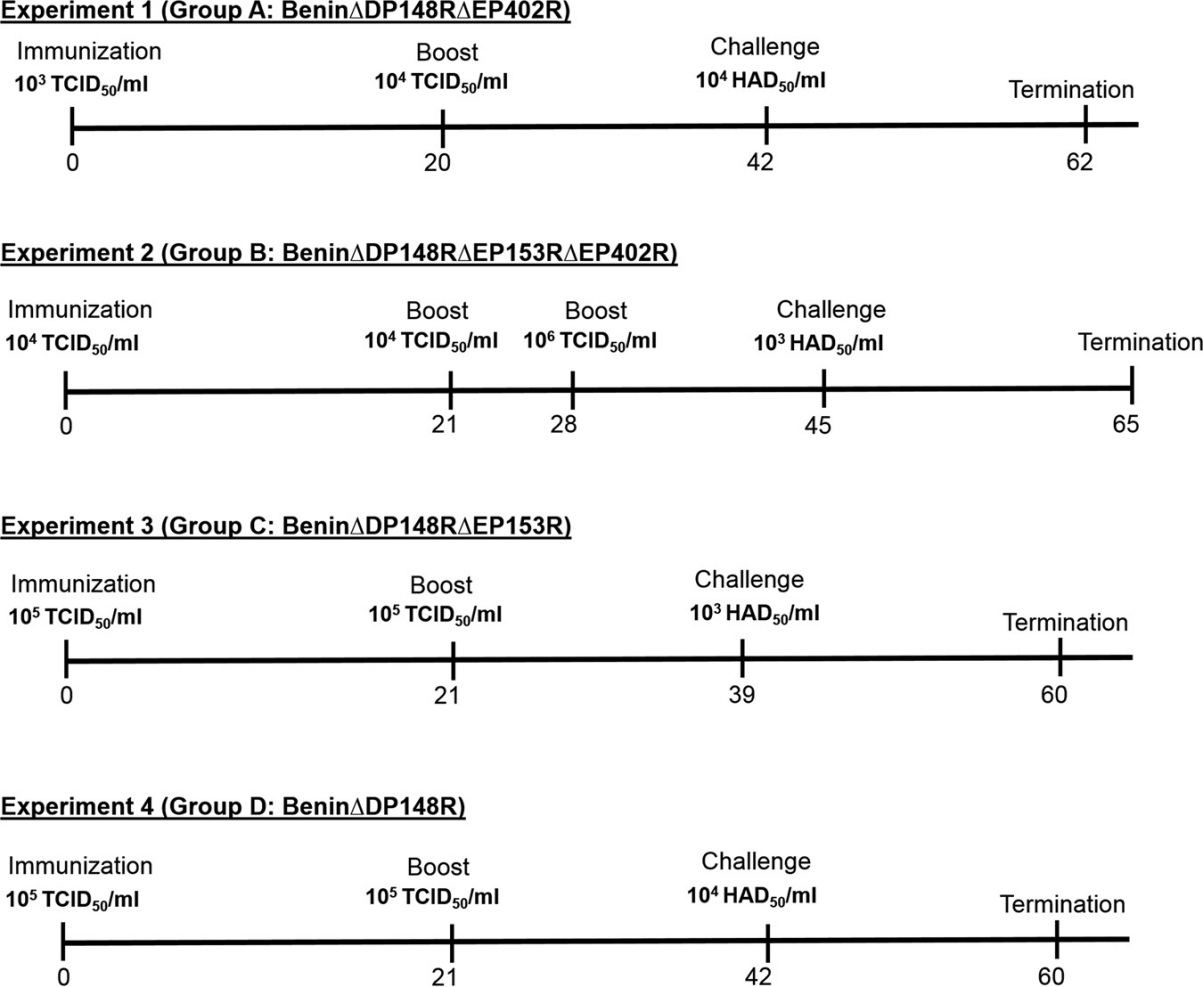
Timeline for the vaccination experiments. The days are given as postimmunization, beginning at day 0 with immunization, followed by boost, challenge with Benin 97/1, and finally termination. The amounts of viruses used are given as either TCID_50_/mL or HAD_50_/mL.

Figure 3 shows the schedule of each of 4 experiments carried out. In experiment 1, we immunized pigs intramuscularly with 1 mL of the double deletion virus BeninΔDP148RΔEP402R (group A) at the same dose of 10^3^ TCID_50_ as we used in the previously published experiments (7) with the single gene deletion BeninΔDP148R. The specific aim of this experiment was to determine if deleting the EP402R gene reduced the lengthy persistence of virus and genome in blood that we observed in the immunization with the single DP148R gene deletion.

In experiment 2, we immunized pigs and boosted at day 21 with 10^4^ TCID_50_ of the triple deletion mutant BeninΔDP148RΔEP153RΔEP402R (group B). The higher dose, compared to that of experiment 1, of 10^4^ TCID_50_ was chosen, as we expected that the triple gene-deleted virus may be more attenuated by the additional deletion of the EP153R gene. An additional boost with 10^6^ TCID_50_ was carried out at day 28, as measurement of cellular responses at day 21 indicated that low levels of ASFV-specific IFN-γ producing cells had been induced and we wanted to increase the chances of inducing a protective response against challenge.

In experiments 3 and 4, we immunized and boosted pigs with 10^5^ TCID_50_ BeninΔDP148RΔEP153R (group C) and 10^5^ TCID_50_ BeninΔDP148R (group D), respectively. The aim of these experiments was to determine the effect of the additional deletion of EP153R from the BeninΔDP148R virus. An additional aim was to determine the safety of both viruses delivered at the higher dose of 10^5^ TCID_50_ compared to the previous dose of 10^3^ TCID_50_.

One pig in group A (A4) was euthanized at day 9 postimmunization due to a non-ASFV-specific condition. Naive, nonvaccinated pigs (groups E, F, L, and M) served as controls for the challenge with the virulent Benin 97/1 isolate. Groups A and E were challenged intramuscularly at 42 days postimmunization (dpi) with 10^4^ 50% hemadsorption dose (HAD_50_) in 1 mL, while group B and group F were challenged at 45 dpi with 10^3^ HAD_50_ in 1 mL with virulent Benin 97/1 virus (Fig. 3). Groups C and L were challenged at 39 dpi, whereas groups D and M were challenged at 42 dpi.

**FIG 4:**
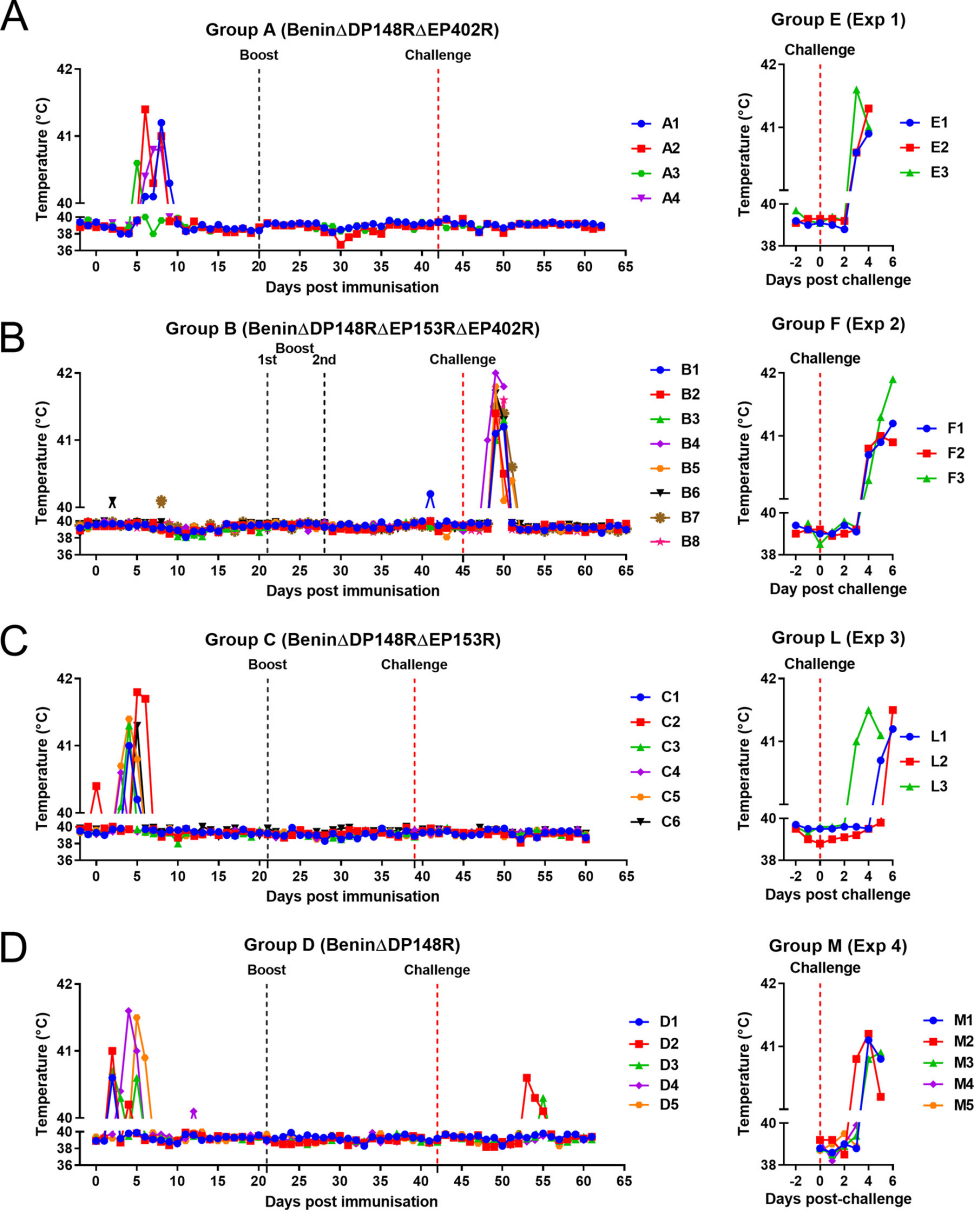
Temperatures following immunization and boost of pigs with BeninΔDP148RΔEP402R (group A), BeninΔDP148RΔEP153RΔEP402R (group B), BeninΔDP148RΔEP153R (group C), and BeninΔDP148R (group D) and challenge with Benin 97/1. Temperatures for nonimmune control pigs after challenge (group E, group F, group L, and group M).

**FIG 5:**
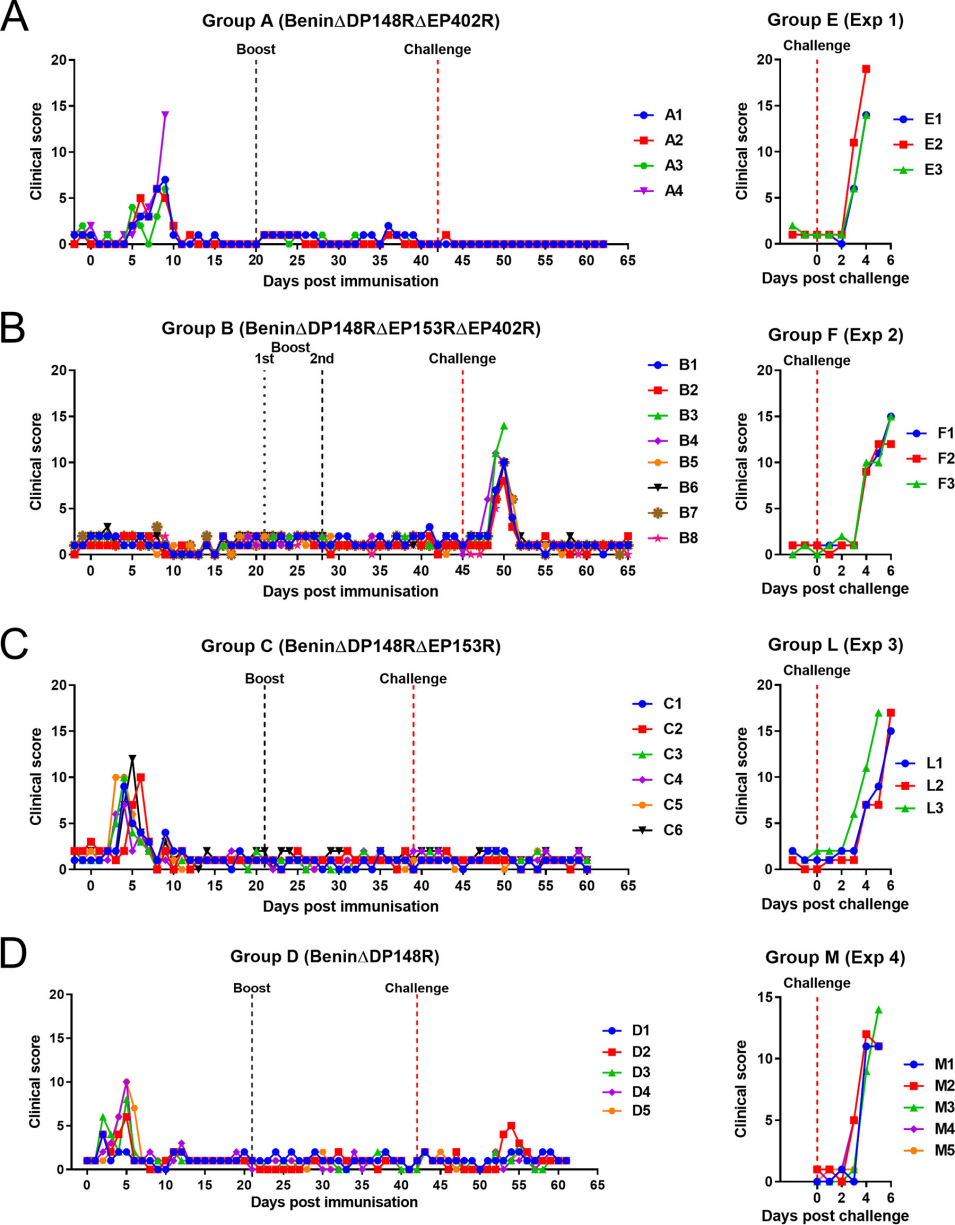
Clinical scores following immunization and boost of pigs with BeninΔDP148RΔEP402R (group A), BeninΔDP148RΔEP153RΔEP402R (group B), BeninΔDP148RΔEP153R (group C), and BeninΔDP148R (group D) and challenge with Benin 97/1. Scores for nonimmune control pigs after challenge (group E, group F, group L, and group M).

Rectal temperatures and clinical scores (28) were recorded daily for all pigs (Fig. 4 and 5). Pigs in group A (BeninΔDP148RΔEP402R) had transient increased temperatures above 40.5°C for 2 days after day 5 postimmunization (Fig. 4A). This was accompanied by reduced appetite and lethargy (Fig. 5A). Pig A1 had a temperature between 40 and 40.5°C for 3 days (6, 7, and 9) and 1 day above 41 (day 8: 41.2). A2 had a temperature at 41°C or above for 2 days (6 and 8) and a temperature of 40.3 on day 7. Pig A3 had a temperature of 40.6°C on day 5 and 40.0 on day 6. One pig, A4, had a temperature of 40.4°C on day 6, 40.8 on days 7 and 8, and 40.0 on day 9. This pig vomited blood and was euthanized on day 9 postimmunization. Postmortem examination showed the pig had a stomach ulcer which was not suspected to be directly related to ASFV infection. No further clinical signs were observed postimmunization in the remaining pigs even after challenge. As expected, the nonimmunized control pigs in group E developed clinical signs associated with acute ASF after challenge. These signs included an increase in temperature (40.6 to 41.6°C), not eating, and lethargy on day 3 postchallenge (Fig. 4A and Fig. 5A). Pig E2 was also vomiting on day 4 postchallenge. All 3 pigs were culled on day 4 postchallenge at the moderate severity humane endpoint.

In group B (BeninΔDP148RΔEP153RΔEP402R), no increase in temperature or other clinical signs were observed in any of the pigs before challenge. After challenge, an increase in temperature at or above 41°C was observed in one pig at 3 days postchallenge and in the remaining pigs at day 4 postchallenge (Fig. 4B). Pigs B3 and B4 also had breathing difficulties on day 5 postchallenge, reaching the humane endpoint, and were euthanized (Fig. 5B). The remaining 6 pigs had an increased temperature above 40.5°C for 2 days in total, except for pig B7, which had a temperature of 40.6°C that persisted for 3 days (Fig. 4B). Nonimmune pigs in group F also developed clinical signs between days 2 and 4 postchallenge, including an increase in temperatures (40.3 to 41.9°C), not eating, and lethargy (Fig. 4B and Fig. 5B). At day 6 postchallenge, pig F2 had hemorrhagic lesions at the periphery of the ears while pig F3 had traces of blood in its feces. On days 4 to 6 postchallenge, all pigs were euthanized at the moderate severity humane endpoint.

In group C (BeninΔDP148RΔEP153R), moderate clinical signs were observed after immunization, comparable with those of group D (BeninΔDP148R), with increased temperatures above 40.5°C for 1 or 2 days between days 3 to 6. However, after challenge, none of the pigs from either of the groups showed any clinical signs or temperatures (Fig. 4C and D and Fig. 5C and D). In clear contrast, the health status of control pig L3 deteriorated quickly starting from day 3 postchallenge, with a raise in rectal temperature above 41.5°C at day 4 postchallenge. Pig L3 was euthanized at day 5 postchallenge, and the rest of the control group L reached the humane endpoint at day 6 postchallenge due to increased temperatures (41.2 to 41.5°C) and rapid manifestation of clinical signs, including lethargy and anorexia (Fig. 4C and Fig. 5C).

In group D (BeninΔDP148R), pigs D1, D2, and D3 had increased temperatures at day 2 postimmunization, with temperatures ranging from 40.6 to 41°C. Pigs D4 and D5 showed increased temperatures on day 4, but these dropped on the following day (Fig. 4D). No temperatures or clinical signs were present after boost or challenge in this group (Fig. 4D and Fig. 5D). Control pigs from group M showed clinical signs, including increased temperature, lethargy, and reduced appetite or anorexia, from day 2 or 3 postchallenge. All were euthanized at day 4 or 5 postchallenge (Fig. 4D and Fig. 5D).

### Postmortem pathological observations.

**FIG 6:**
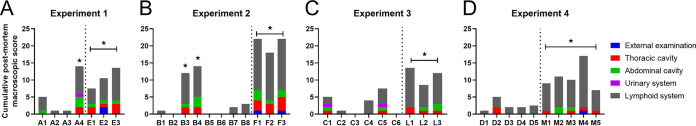
Scoring macroscopic lesions at the cull point for the vaccinated groups, experiment 1, BeninΔDP148RΔEP402R (group A), experiment 2, BeninΔDP148RΔEP153RΔEP402R (group B), experiment 3, BeninΔDP148RΔEP153R (group C), and experiment 4, BeninΔDP148R (group D), and the control groups (E, F, L, and M). Lesions are presented on the graph by different colors. Stars represent the animals that reached the endpoint before study termination.

Apart from mild lymphadenopathy, pigs in group A (A1, A2, and A3) immunized with BeninΔDP148RΔEP402R did not show any other relevant macroscopic lesions. In this group, pig A4 was euthanized due to welfare reasons before challenge, not displaying lesions characteristic of ASF (Fig. 6). In group B, immunized with BeninΔDP148RΔEP153RΔEP402R, the pigs culled at humane end points (B3, B4) had macroscopic lesion scores higher than those of pigs which survived but lower than those of the control pigs. Pigs B3 and B4 displayed characteristic ASF lesions, such as mild to moderate ascites and mild to moderate hyperemic splenomegaly, along with lymph nodes displaying lymphadenitis and petechial hemorrhages. In some lymph nodes, including the renal lymph nodes, hemorrhages were so severe that lymph nodes seemed like blood clots (hemorrhagic lymphadenitis). Three surviving pigs (B1, B7, and B8) had enlarged lymph nodes, while the other three (B2, B5, and B6) were free of any ASF-typical lesions (Fig. 6). In group C, immunized with BeninΔDP148RΔEP153R, pigs C1 and C5 showed the highest lesion scores displaying mild hyperemic splenomegaly and mild lymphadenitis with occasional petechial hemorrhages that affected mainly the renal and gastrohepatic lymph nodes (Fig. 6). Similar findings were observed in group D (BeninΔDP148R), where pigs D1, D3, D4, and D5 showed enlarged lymph nodes. Pig D2 also displayed mild hyperemic splenomegaly and mild hydropericardium. (Fig. 6). All control pigs belonging to groups E, F, L, and M presented lesions consistent with acute ASF, characterized by erythematous tonsils, hydropericardium, enlarged spleens with friable consistency, ascites, and generalized lymphadenitis with petechial hemorrhages, that in some lymph nodes (mainly gastrohepatic and renal) became more severe (hemorrhagic lymphadenitis).

### Infectious virus and genome in blood.

**FIG 7:**
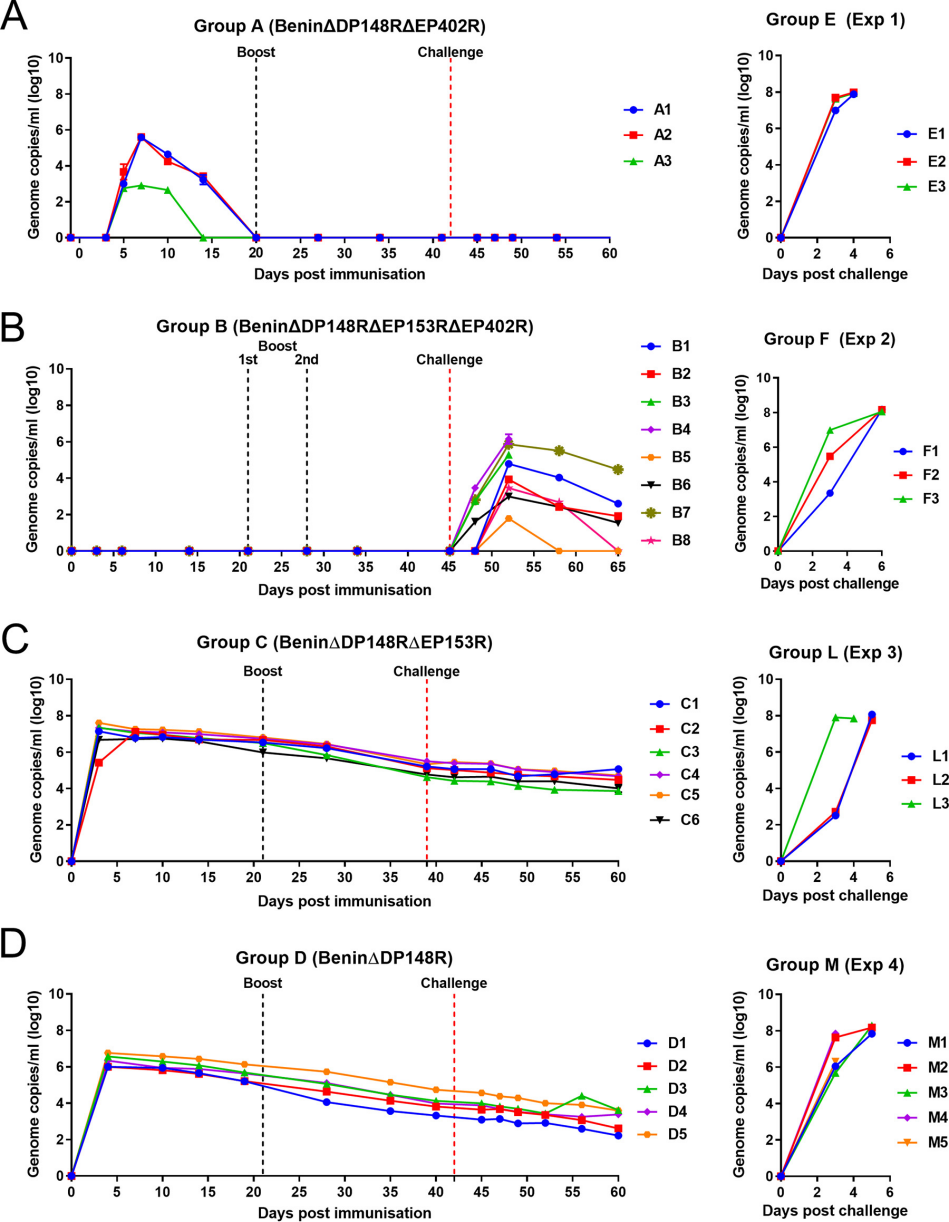
Levels of viral genome in blood of pigs immunized with BeninΔDP148RΔEP402R (group A), BeninΔDP148RΔEP402RΔEP153R (group B), BeninΔDP148RΔEP153R (group C), BeninΔDP148R (group D). Levels of control groups corresponding to each study are presented in the right panels (groups E, F, L, M). Results are estimated by qPCR and reported as genomic copies/mL (log_10_) of blood.

Levels of virus genome in blood were measured by qPCR and infectious virus by titration in porcine bone marrow cells. In group A (BeninΔDP148RΔEP402R), virus genome was first detected at day 5 postimmunization, coinciding with the onset of clinical signs, and increased to a peak of between 10^5^ to 10^6^ genome copies per milliliter in pigs A1 and A2 and 10^3^ in pig A3 (Fig. 7A). Virus genome levels decreased after day 10 or 14 postimmunization and were not detectable by day 20. No pigs had detectable virus genome after boost and challenge.

**FIG 8:**
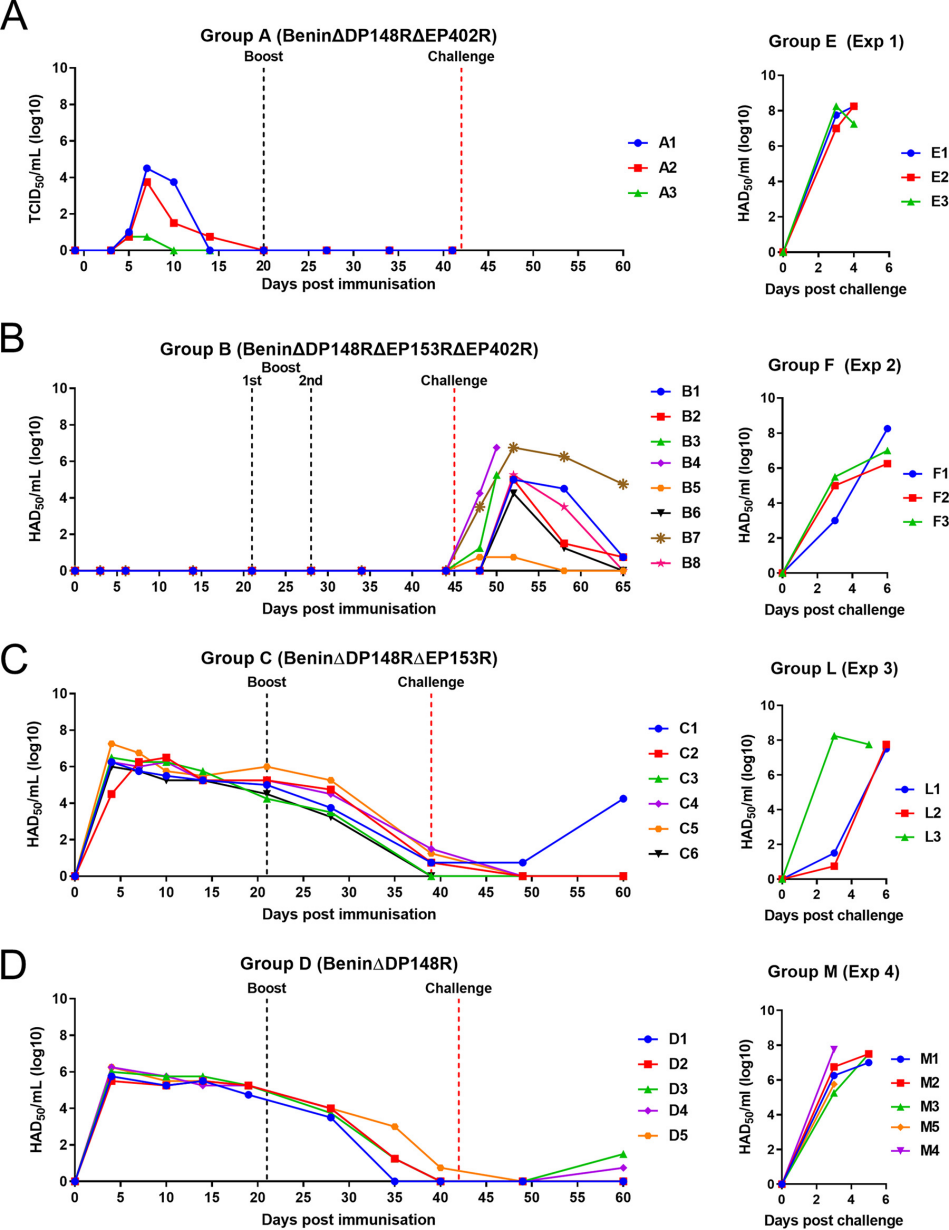
Levels of infectious virus in blood of pigs immunized with BeninΔDP148RΔEP402R (group A), BeninΔDP148RΔEP153RΔEP402R (group B), BeninΔDP148RΔEP153R (group C), BeninΔDP148R (group D). Levels in control groups corresponding to each study are presented in the right panels (groups E, F, L, M). Virus titers are shown as HAD_50_/mL for groups B, C, and D and control groups E, F, L, and M. For group A, values are shown as TCID_50_/mL.

Titration of infectious virus from blood showed a similar pattern, although detection was lower than that of genome copies (Fig. 8A). Infectious virus was first detected at day 5 postimmunization and by day 7 increased to a peak of 10^3.75^ to 10^4.5^ per milliliter in pigs A1 and A2. In pig A3, infectious virus was detected at a level below accurate measurement. Infectious virus was not detected by day 14 postimmunization in A1 and A3, and only very low levels were detected in pig A2 at this time. No infectious virus was detected after boost or challenge.

In group B (BeninΔDP148RΔEP153RΔEP402R), no virus genome was detected in blood from pigs before challenge (Fig. 7B). After challenge, variable levels of genome were detected from day 3 or 4 postchallenge. The highest levels were detected in pigs B3 and B4, which reached the humane endpoint. At termination, values in these pigs were between 10^5.3^ and 10^6.2^ genome copies per milliliter. In the surviving 6 pigs, the maximum levels detected varied between ∼10^2^ and 10^5.9^ genome copies/mL. Levels of virus genome decreased until termination at day 20 postchallenge, becoming undetectable in 2 pigs (B5 and B8) and reduced to 10^2.6^ in others (B1, B2, and B6). Only in one pig (B7), levels remained at 10^4.5^ at termination.

In group B, no infectious virus was detected in blood before challenge even when the volume added to titrations was increased 10-fold. After challenge, levels of infectious virus were detected at day 3 postchallenge in pigs B3 and B4 and reached levels at termination of 10^5.25^/mL in pig B3 and 10^6.75^/mL in pig B4 (Fig. 8B). In the other pigs, which survived challenge, levels of infectious virus varied and were highest in pig B7 (10^6.75^/mL) at day 7 postchallenge, reducing to 10^4.75^/mL at the end of the experiment. The peak infectious virus after challenge in the other pigs varied between 10^4.25^ and 10^5.25^, except that for pig B5, which had infectious virus below the level of accurate measurement. At termination of the experiment, very low or no infectious virus was detected in these pigs.

Pigs from group C (BeninΔDP148RΔEP153R) had higher levels of genome copies in blood (approximately 10^6.7^ to 10^7.6^ genome copies/mL) at 3 days postimmunization for all pigs except C2, in which levels peaked at day 7. These values then declined gradually but persisted throughout the experiment and had decreased to between 10^4^ to 10^5^ genome copies/mL by the end of the study (day 60) (Fig. 7C). Levels of infectious virus (Fig. 8C) in blood peaked in all pigs except C2, at day 3 postimmunization between 10^6^ and 10^7.25^ HAD_50_/mL. In pig C2, infectious virus peaked at 10^6.5^ HAD_50_/mL by day 10 postimmunization. Levels of infectious virus declined, becoming undetectable or at very low levels by day 39 postimmunization. However, in one pig, C1, infectious virus dropped to very low levels after challenge but increased at termination to 10^4.25^ HAD_50_/mL. Since this virus expressed the fluorescent protein markers, we concluded it was the BeninΔDP148RΔEP153R used for immunization.

Pigs in group D (BeninΔDP148R) had between 10^6^ to 10^6.8^ genome copies per milliliter by day 4 postimmunization, and thereafter levels of genome decreased gradually to between 10^2^ to 10^3.6^ genome copies/mL at termination on day 60 (Fig. 7D). Levels of infectious virus in blood in group D (Fig. 8D) also peaked at day 4 postimmunization between 10^5.5^ to 10^6.25^ HAD_50_/mL and dropped to become undetectable by day 35 or 40 postimmunization. At termination, infectious virus was detected at levels too low to measure accurately in just two pigs, D3 and D4.

Levels of genome copies in blood at day 10 postimmunization for group C were significantly higher (*P* < 0.01) than those for group D, and this difference persisted throughout the study (*P* < 0.1 at days 14, 28, 39/40, and 49 postimmunization). However, no significant differences were detected in levels of infectious virus comparing groups C and D.

As expected, very high levels of virus genome and infectious virus (10^7^ to 10^8^ per milliliter) were detected in all pigs from each control group (groups E, F, J, M).

### IFN-gamma ELISpot assay.

**FIG 9:**
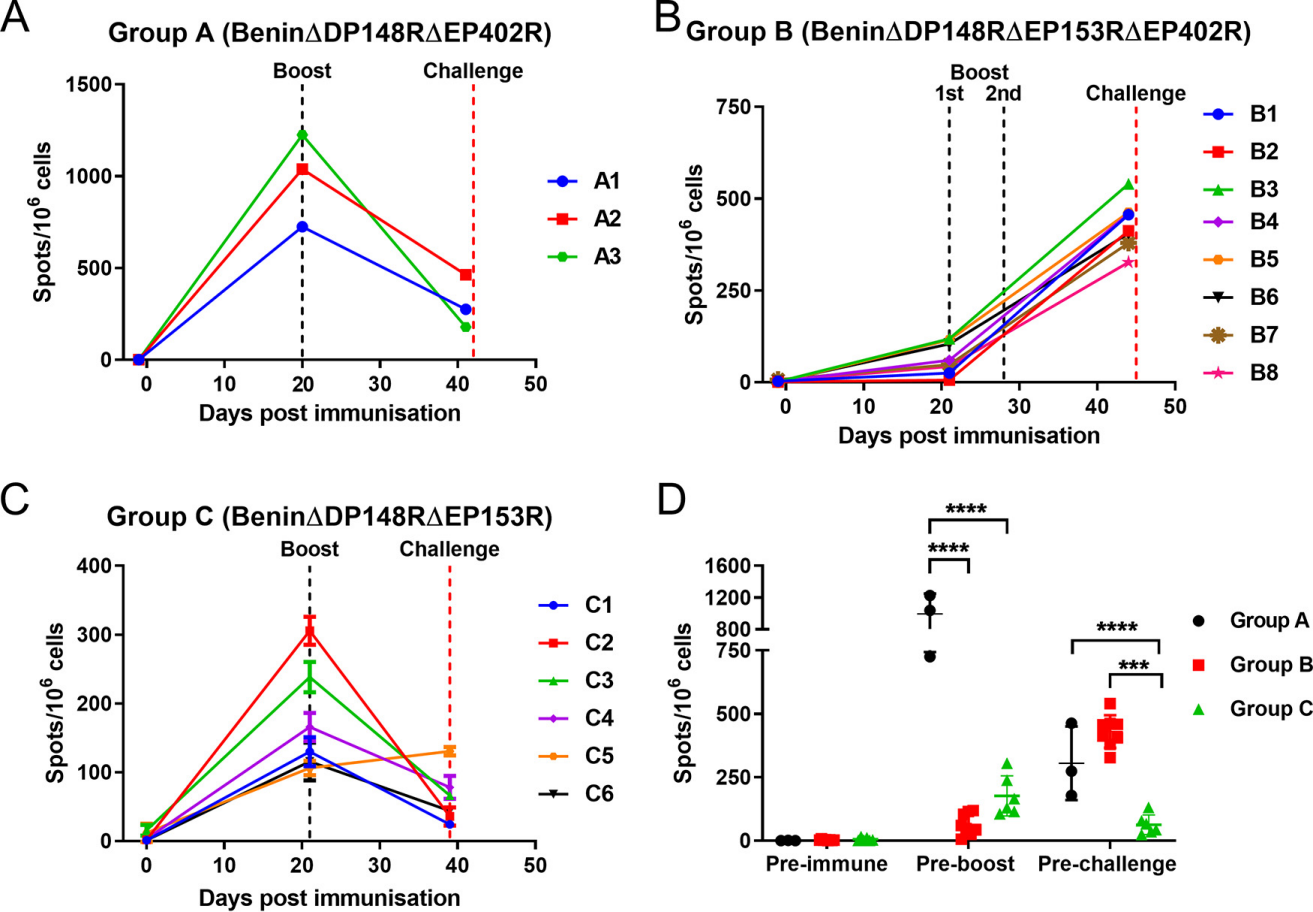
IFN-γ responses from PBMCs collected postimmunization, pre-boost, and before challenge. Panels A, B, and C show the number of IFN-γ-secreting cells stimulated with Benin 97/1 by ELISpot assay. PBMCs were collected from pigs immunized with BeninΔDP148RΔEP402R (group A), BeninΔDP148RΔEP153RΔEP402R (group B), and BeninΔDP148RΔEP153R (group C). Results are presented as mean number of IFN-γ-producing cells/10^6^ cells. Statistically significant responses between groups preboost and prechallenge are presented in panel D (****, *P* < 0.0001; ***, *P* < 0.001).

The responses of peripheral blood mononuclear cells (PBMCs) from immunized pigs to ASFV were measured at different times postimmunization by IFN-γ enzyme-linked immunosorbent spot (ELISpot) assay. An ASFV-specific response was not detected before immunization in any group of pigs. Very high numbers of IFN-γ-producing cells (∼725 to 1,225 spots per million cells) were induced in all pigs in group A before boost, which then decreased before challenge to levels ranging from 178 to 463 spots/million cells (Fig. 9A). In contrast, for group B, low levels of IFN-γ-producing cells were induced after immunization and before the first boost. Hence, a second boost with a higher dose of the same virus was applied 4 weeks postimmunization and the number of IFN-γ-producing cells increased uniformly in all pigs before challenge (326 to 540 spots/million PBMCs) (Fig. 9B). Of note, pigs B3 and B4, which were euthanized at 5 days postchallenge, had 540 and 458 spots/million PBMC. Pigs in group C showed a relatively lower number of IFN-γ-producing cells before boost, with pig C2 showing a higher response of ∼305 spots/million PBMCs, compared with those of other pigs of this group. Surprisingly, levels decreased after the boost and only one pig (C5) had a number of IFN-γ-secreting cells (135 to 126 spots/million PBMCs) at challenge higher than that before boost (Fig. 9C). The number of IFN-γ-producing cells was significantly higher in group A compared with that in groups B and C before boost. At challenge, both groups A and B showed a number of IFN-γ-producing cells significantly higher than that of group C (Fig. 9D).

### Antibody responses.

**FIG 10:**
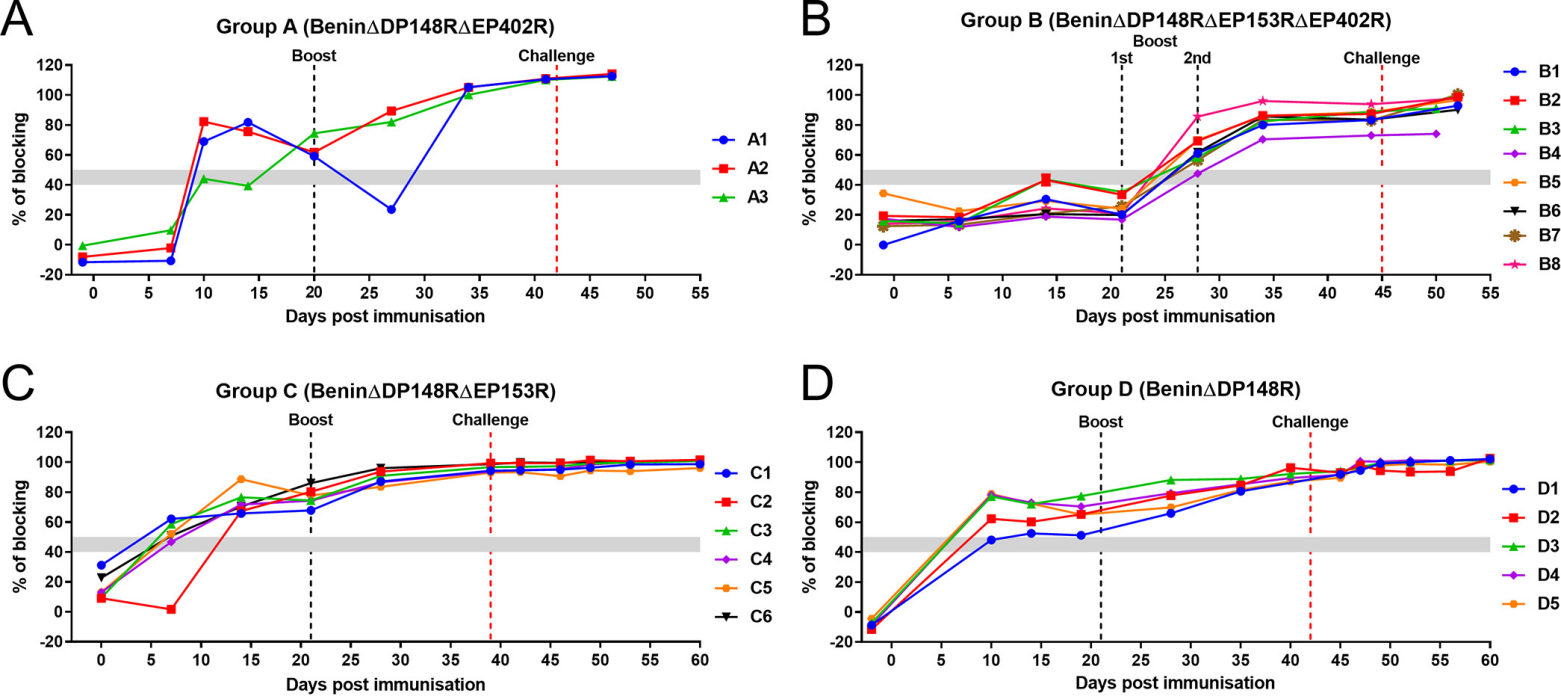
Antibody responses postimmunization. Serum samples were collected from pigs immunized with BeninΔDP148RΔEP402R (group A), BeninΔDP148RΔEP153RΔEP402R (group B), BeninΔDP148RΔEP153R (group C), and BeninΔDP148R (group D) and assayed using a commercial blocking ELISA against p72 protein. Results are presented as percentage of blocking and cutoff value is represented by 50%.

Antibody responses to ASFV p72 capsid protein were measured using a commercially available blocking ELISA. As expected, an antibody response was detected in all pigs in group A by day 14 and increased after boost (Fig. 10A). In contrast, pigs in group B mounted a slower antibody response, which was first detected at day 27 postimmunization after the first boost and just before the second boost. Levels were maintained for the rest of the experiment (Fig. 10B). A faster antibody response was seen in group C, where most of the pigs had detectable antibody by day 7, and on day 14 all were above the cutoff (Fig. 10C). For group D, a general trend was observed in generating an antibody response at day 10 (Fig. 10D).

## DISCUSSION

Targeted deletions of nonessential genes from the ASFV genome have been used successfully to construct candidate live attenuated vaccine strains and to understand the role of the genes during infection of cells and pigs. A vaccine candidate with an acceptable safety profile should have reduced clinical signs and vaccine virus persistence postimmunization but retain high levels of protection.

One of the potential candidates for targeted deletions represents the EP402R gene, which encodes the CD2v protein, since it is required for the binding of infected cells or extracellular viral particles to red blood cells, thus playing an important role in viral dissemination in different tissues in pigs. During the sylvatic cycle, in ticks, the binding of the virus to red blood cells probably helps retention of the virus with the blood meal and also crossing the midgut barrier (29). Deletion of the EP402R gene from the genome of virulent viruses Malawi Lil20/1 (genotype VIII) (16) and Georgia/07 (genotype II) (12) did not reduce the virulence of these viruses in pigs. Surprisingly, deletion of this gene from a genotype I virulent isolate, BA71, reduced the virus virulence and induced protection against lethal challenge with the parental virus and also against the genotype II Georgia/07 isolate (30). Recent studies have shown that deletion of an additional virulence marker DP96R (UK) gene from a genotype II EP402R/CD2v gene-deleted Chinese isolate (ASFV-SY18) resulted in attenuation in pigs, conferring 100% protection after challenge with the homologous virulent strain. However, viral DNA was still present in different lymphoid organs after the challenge (31). Deletion of the EP153R gene from the genome of a virulent isolate, Malawi Lil20/1 (genotype VIII), failed to reduce virulence of the virus for pigs (32). Both the EP402R and EP153R genes are interrupted in the genomes of the naturally attenuated isolates OURT88/3 and NH/P68 (33, 34). However, the role of these gene interruptions in virus virulence is unclear since OURT88/3 and NH/P68 viruses also have large deletion of multiple genes belonging to MGF 360 and MGF 505 from close to the left genome end (33, 35). Both the single deletion of EP402R and the simultaneous deletion of EP402R and EP153R from a gene-deleted attenuated Georgia/07 strain (ASFV-G-Δ9GL) decreased the ability to protect against challenge (36). Thus, it is apparent that additional genes deleted in combination with the EP402R gene may result in virus attenuation and, in some examples, induction of protection. Since EP402R and EP153R are conserved in most virulent isolates, we expect that their deletion, in combination with other virulence factors, should have a similar effect in different genotypes. Serum antibodies against both CD2v and EP153R were shown to mediate hemadsorption inhibition and to be involved in serotype-specific protective immunity, representing good targets for ASFV serotype classification and evolution (37, 38).

Our approach to reduce the virus persistence and clinical signs postimmunization of pigs with a moderately attenuated ASFV isolate, BeninΔDP148R (7), was to remove EP402R or EP153R genes either singly or in combination from the BeninΔDP148R attenuated virus. The results of our current and previous experiments are summarized in Table 1. Previously, we showed that, following immunization of pigs with BeninΔDP148R at 10^3^ HAD_50_, virus genome levels peaked by day 5 or 6 postimmunization and gradually declined but persisted until the end of the experiment at day 59. Infectious virus also peaked at day 5 or 6 postimmunization but declined more rapidly and was not detected after day 28 postimmunization. Similar results were obtained here using a higher dose of 10^5^ HAD_50_/mL of the BeninΔDP148R virus (group D) and 10^5^ HAD_50_ mL of the BeninΔDP148RΔEP153R virus (group C). In both groups C and D, a moderate level of virus genome in blood was observed to peak early postimmunization, coincident with the onset of clinical signs, and gradually declined, although levels of genome of 10^2^ to 10^4^ per milliliter were detected at termination on day 59 or 60 postimmunization. Levels of infectious virus were similar to genome levels at 3 or 4 days postimmunization but then declined more rapidly, and no or very low levels of infections virus were present by day 39 or 40 postimmunization, a few days before challenge. These results are consistent with an early virus replication in blood coinciding with clinical signs. The gradual decline of virus levels in blood thereafter is consistent with our hypothesis that virus persists attached to the red blood cells mediated by the CD2v protein on the surface of extracellular virus particles. The more rapid decline of infectious virus in comparison to that of genome may be due to a loss in infectivity of the nonreplicating virus attached to red blood cells over time. Comparison of immunizations with 10^3^ or 10^5^ HAD_50_ of BeninΔDP148R showed that peak virus genome and infectious virus levels after immunization were similar as were clinical signs after immunization and challenge. The time to the onset of clinical signs was reduced by 1 or 2 days following immunization with the higher dose.

**TABLE 1:** Summary of results on virus and genome persistence in blood^*a*^

Virus	Dose (TCID_50_)	Peak viremia PI (avg)		Duration of viremia (dpi)		Fever PI		
		Genome copies/mL (log10)	Infectious virus (log10)	Genome	Virus	No. pigs	Peak (avg) (°C)	Duration (days)
BeninΔDP148R	10^3^, 10^3^	6.2	6.1	4–59	4–28	5/5	41.0	1–2
BeninΔDP148RΔEP402R	10^3^, 10^4^	4.7	3	5–14	5–14	4/4	41.1	1–2
BeninΔDP148RΔEP153RΔEP402R	10^4^, 10^4^, 10^6^	None	None	None	None	0/8	None	None
BeninΔDP148RΔEP153R	10^5^, 10^5^	7.2	6.5	3–60	3–39	6/6	41.2	1–3
BeninΔDP148R	10^5^, 10^5^	6.3	6.0	4–60	4–40	5/5	41.1	1–2

a The virus used for immunization is shown in column A, and the dose used for prime and boost is shown in column B. The peak viremia postimmunization (PI) measured as genome copies per milliliter or infectious virus in blood is shown in column C, and the duration of these in days postimmunization (dpi) is shown in column D. Column E shows the peak fever postimmunization in degrees Celsius, the number of pigs showing fever of 40.6°C or above, and the duration of fever in days.

In contrast to these results, from the immunizations of pigs with BeninΔDP148R and BeninΔP148RΔEP153R, deletion of the EP402R gene from the BeninΔDP148R virus resulted in a much shorter period of virus persistence in blood. Immunization of pigs with the virus BeninΔDP148RΔEP402R (group A) resulted in detection of both virus genome and infectious virus in blood only until day 14 postimmunization (Table 1, Fig. 7A, and Fig. 8A) (7). These results support our hypothesis that CD2v plays a role in viral persistence in blood by mediating binding of extracellular virus particles to red blood cells. This may shield the virus particles to protect them from clearance by immune cells.

The group A immunized pigs still displayed moderate clinical signs postimmunization and mounted a strong immune response since all pigs were protected against challenge with the virulent virus. We were surprised that deleting the EP402R gene did not have a greater effect on reducing clinical signs postimmunization, since attenuation was observed when this gene was singly deleted from the virulent BA71 virus, another genotype I isolate (30). In order to try to further decrease clinical signs after immunization with BeninΔDP148RΔEP402R, we also deleted the EP153R gene, which is adjacent to EP402R (33). This gene encodes a type II membrane protein containing a C-type lectin domain similar to those in host proteins. The EP153R protein has been shown to have diverse roles, including increasing the binding of red blood cells to ASFV-infected cells, inhibition of cell surface expression of SLAI (porcine major histocompatibility complex [MHC] class I), and inhibition of apoptosis (23–25). It was also shown that together with CD2v, EP153R can contribute to mediating the cross-protective serotype-specific immunity (38).

After immunization with BeninΔDP148RΔEP153RΔEP402R (group B), pigs did not show any clinical signs or viremia before challenge (Fig. 4B, Fig. 5B, Fig. 7B, and Fig. 8B). However, as discussed above, when we deleted EP402R or EP153R singly, elevated clinical signs were observed after immunization. This could be explained if the proteins act synergistically and thus the deletion of both genes reduces the viral load to an extent that the innate immune response can suppress the initial steps of the viral replication. A faster antibody response was mounted in groups A (BeninΔDP148RΔEP402R), C (BeninΔDP148RΔEP153R), and D (BeninΔDP148R), whereas pigs in group B (BeninΔDP148RΔEP153RΔEP402R) showed a slower antibody response, since the animals seroconverted only after the second boost (Fig. 10). The cell-mediated immune responses were measured by stimulating PBMCs with ASFV and measuring the numbers of IFN-γ-producing cells by ELISpot assays. PBMCs collected from group A (BeninΔDP148RΔEP402R) had responses very much higher than those collected from group B (BeninΔDP148RΔEP153RΔEP402R) and group C (BeninΔDP148RΔEP153R) before the boost (Fig. 9D). A previous publication showed that deletion of the EP402R gene from a virulent isolate abrogated the inhibition of lymphocyte proliferation in response to mitogens observed following infections of PBMCs *in vitro* (16). An intriguing possibility is that our observations may result from the removal of an inhibitory effect of CD2v on lymphocyte function following infection of pigs with the BeninΔDP148RΔEP402R. Possibly, expression of the CD2v protein may inhibit lymphocyte functions *in vivo* as well as *in vitro*. The results might also be explained if reduced binding of red blood cells to infected cells enhanced recognition by the immune system of virus antigens presented to T cells. This may explain the enhanced cellular response we observed. The differences may also result from the impact of virus infection on PBMCs and hence on the ELISpot results. Since PBMCs were purified using histopaque gradients, we would expect infected monocytes but only trace amounts of extracellular virus to be present. The cellular composition and activation of the PBMC may vary depending on the impact of the different viruses. For example, various levels of infection in blood may differentially affect antigen presentation or release of factors such as cytokine and chemokines. Further research is needed to resolve the mechanisms involved.

Interestingly, PBMC from pigs immunized with BeninΔDP148RΔEP153RΔEP402R had a considerable cellular response only after boost (Fig. 9B). A statistical significance was not observed between numbers of IFN-γ-producing cells stimulated by ASFV in PBMCs from groups A and B before challenge (Fig. 9D). After challenge, pigs in group B (BeninΔDP148RΔEP153RΔEP402R) presented moderate viremia (up to 10^6^ genome copies/mL) (Fig. 7B), with 2 pigs reaching the endpoint. This indicates that the immune response induced was not sufficient to suppress replication of the challenge virus. As discussed above, BeninΔDP148RΔEP402RΔEP153R possibly did not replicate to the same extent, as no viremia was detected after immunization or boost, and consequently a reduced immune response was induced. Since all viruses replicated to a level similar to that of parental virus in macrophages *in vitro*, the reduced replication in blood *in vivo* of BeninΔDP148RΔEP153RΔEP402R must have resulted from interactions with host factors.

In summary, we showed that by removing EP402R from the BeninΔDP148R backbone, we reduced viral persistence in blood after immunization but maintained a survival rate of 100% after challenge and no replication of challenge virus. The additional deletion of EP153R increased attenuation, since no clinical signs or viremia were observed after immunization; however, elevated clinical signs and viremia were observed after challenge with 75% survival rate. Deletion of EP153R alone did not reduce virus persistence or clinical signs after immunization compared to the single deletion of DP148R gene. These results highlight an important role for both EP153R and EP402R proteins acting synergistically to control levels of virus replication and persistence in blood *in vivo*. This may be mediated by the cooperation of both proteins in binding of virus particles and infected cells to red blood cells and possibly other cells. In addition, the two proteins may also act singly or synergistically to evade initial steps of the immune response. A role in immune modulation has already been shown for the EP402R protein. C-type lectin proteins have diverse roles in mediating cell-to-cell adhesion. For example, C-type lectin receptors play a crucial role in natural killer (NK) cell activity. It is tempting to speculate that EP153R may have evolved to evade NK host immune responses since NK activity was shown to correlate with protection following immunization with the attenuated NH/P68 isolate (35).

Overall, the viruses we have constructed from already attenuated virus provide the means to dissect the role of the EP153R and EP402R/CD2v proteins and other viral proteins in pathogenesis and evasion of immune responses. Although the results of our study are specific to the genotype I BeninΔDP148R virus, we expect that results could be transferred to other strains and genotypes of virus. However, further work will be required to confirm this. Deletion of the EP402R gene to reduce the period of virus persistence in blood, while maintaining induction of a strong immune response, would be a highly valuable phenotype for candidate vaccine strains to limit transmission and spread in the environment of the vaccine. The development of ASFV vaccines with potential to cross-protect between genotypes would be desirable, particularly in regions where more than one genotype is circulating. It would therefore be important in future to test the ability of attenuated genotype I strains to induce cross-protection against other circulating genotypes, particularly against genotype II strains.

## MATERIALS AND METHODS

### Viruses and cells.

The ASFV Benin 97/1 wild-type and BeninΔDP148R isolates have been described elsewhere (7, 33). Deletion mutant viruses were cultured in porcine bone marrow cells (PBMs). Titration of wild-type virus was carried out in 96-well plates seeded with PBM by hemadsorption assay (in which the results are presented as HAD_50_/mL) or endpoint titers based on expression of mNeonGreen or TagRFP-T fluorescent protein markers and presented as TCID_50_/mL, calculated using the Spearman and Karber formula. To increase the sensitivity of virus detection, blood samples with no detectable infectious virus by titration were further tested by adding 100 μL of the sample to 6-well plates seeded with PBMs.

### Construction of recombinant BeninΔDP148RΔEP402R, BeninΔDP148RΔEP153RΔEP402R, and BeninΔDP148RΔEP153R.

**Transfer plasmids.** Transfer plasmid pΔEP402R-VP72GUS was constructed by cloning amplified right and left regions of the Benin 97/1 flanking the EP402R gene into the previously published pLoxPVP72GUSLoxP vector (39).

Vector pLoxPVP30TagRFP-TLoxP was generated by first amplifying the TagRFP-T gene (40) with a forward primer containing BamHI restriction site (italic) and the ASFV P30 promoter sequence (bold) (41) (5′*-G**GATCC***TTATTATTTTATAATTTTAAAATTGAATGGATTTTATTTTAAATATATCC**ATGGTGTCTAAGGGCGAAGAGCT) and a reverse primer containing an ASFV transcription termination signal (bold) (42) and EcoRI restriction site (italic) (5′-*GAATTC***AAAAAAAAAA**CTTGTACAGCTCGTCCATGCCAT). The TagRFP-T fluorescent marker was kindly provided by Chris Netherton (The Pirbright Institute, UK). The amplified product was then swapped with the VP72GUS cassette of pLoxPVP72GUSLoxP vector. Using this newly produced pLoxPVP30TagRFP-TLoxP, the left and flanking regions of DP148R were then cloned to produce transfer plasmid pΔDP148R-VP30TagRFP-T.

The other transfer plasmids pΔEP153R-VP30mNG and pΔEP153RΔEP402R-VP30mNG were synthesized commercially (Genscript, USA). pΔEP153R-VP30mNG contains the left and right flanking regions of EP153R, while pΔEP153RΔEP402R-VP30mNG contains the left flanking region of EP153R and the right flanking region of EP402R. In both plasmids, between the flanking regions, a reporter gene, mNeonGreen (mNG), under the control of the ASFV VP30 promoter flanked with LoxP sites was added (26).

**Homologous recombination and virus purification.** Recombinant ASFV BeninΔDP148RΔEP402R was produced in a sequential 2-step deletion method. First, BeninΔEP402R (Fig. 1B) was produced by infecting primary porcine alveolar macrophages (PAM) with Benin 97/1 (Fig. 1A) and then transfection with pΔEP402R-VP72GUS using the TransIT-LT1 transfection reagent (Mirus Bio, USA). In the presence of X-Gluc, recombinant viruses expressing the GUS gene were identified and purified by multiple rounds of limiting dilutions. Next, using purified BeninΔEP402R as parental virus, homologous recombination was undertaken with transfer plasmid pΔDP148R-VP30TagRFP-T in wild boar lung cells (WSL-R). Cells expressing the TagRFP-T were identified via fluorescence-activated cell sorting (FACS); single cells were isolated and cultured in individual wells of 96-well plates containing PBMs. The recombinant virus was subsequently purified by FACS using the method described by Rathakrishnan et al. (26). Similarly, to produce recombinants BeninΔDP148RΔEP153R (Fig. 1C) and BeninΔDP148RΔEP153RΔEP402R (Fig. 1D), WSL-R cells were infected with the BeninΔDP148R virus at a multiplicity of infection (MOI) of 2 and transfected with the pΔEP153R-VP30mNG or pΔEP153RΔEP402R-VP30mNG plasmids, respectively, using the TransIT-LT1 transfection reagent. Expression of mNeonGreen marker was monitored and recombinant viruses were isolated and purified by FACS and limiting dilution as described previously (26). Sequencing across the site of deletion confirmed the expected deletion and site of reporter gene insertion.

### Growth curves.

Viruses (Benin 97/1, BeninΔDP148R, BeninΔDP148RΔEP402R, BeninΔDP148RΔEP153RΔEP402R, and BeninΔDP148RΔEP153R) were added to purified PBMs at an MOI of 0.01 in triplicate in 24-well plates. Cells and supernatants were collected at different times postinfection and subjected to 3 freeze-thaw cycles. Cellular debris was removed by centrifugation, and virus titers were determined by the fluorescence assay or HAD, as described above. The experiment was carried out in purified PBMs from 2 different pigs.

### Immunization and challenge of pigs.

Animal experiments were carried out in SAPO4 high-containment animal housing at the Pirbright Institute according to regulated procedures from the Animals Act UK 1998 and conducted under Home Office License 7088520. Four separate experiments were undertaken. In experiment 1, 4 large white X Landrace pigs (15 to 20 kg) were immunized by the intramuscular route with 10^3^ TCID_50_ BeninΔDP148RΔEP402R and boosted by the same route with the same recombinant virus at 10^4^ TCID_50_ on day 20 postimmunization. In experiment 2, 8 pigs were immunized by the intramuscular route with 10^4^ TCID_50_ BeninΔDP148RΔEP153RΔEP402R and were boosted twice with the same virus at 10^4^ TCID_50_ on day 21 and at 10^6^ TCID_50_ on day 28 postimmunization. The additional boost with 10^6^ TCID_50_ was performed following the measurement of both cellular and antibody responses at day 21, which indicated low levels of ASFV-specific antibodies and that IFN-γ-producing cells had been induced. In experiment 3, 6 pigs were immunized by the intramuscular route with 10^5^ TCID_50_ BeninΔDP148RΔEP153R and boosted with the same dose on day 21 postimmunization. In experiment 4, 6 pigs were immunized by the intramuscular route with 10^5^ TCID_50_ BeninΔDP148R and boosted with the same dose on day 21 postimmunization. Nonimmunized control pigs and immunized pigs were challenged by the intramuscular route with either 10^4^ (experiment 1, group E and for experiment 4, group M) or 10^3^ (experiment 2, group F, for experiment 3, group L) HAD_50_ of the virulent Benin 97/1 isolate approximately 3 weeks after the boost (Fig. 3).

Daily clinical scoring was carried out to record temperatures, anorexia, behavior including lethargy, vomiting, or hemorrhagic diarrhea, respiratory distress, lameness, and hemorrhagic skin lesions (28). Pigs were euthanized at a moderate severity endpoint as defined in the project license PPL70/8852 from the UK Home Office. The moderate humane endpoint was typically reached following 4 days of temperature at 40.6°C or above or for 3 days if other clinical signs were observed. Other signs contributing to the endpoint included not eating for a second day and unwillingness to get up. Occasionally, other signs were observed. At necropsy, enlargement and appearance of hemorrhages in spleen, tonsils, and lymph nodes were recorded in addition to other signs, including lung pathology, petechiae on kidneys, fluid in the pericardium, or ascites (43).

### Genome copies in blood.

DNA was extracted from whole blood using MagVet universal isolation kit (Life Technologies) at different days throughout the study. Samples were assayed in duplicate for the presence of viral DNA by quantitative PCR (qPCR) on a Stratagene Mx3005P system (Agilent Technologies, Santa Clara, CA, USA) following a protocol modified (44) from using the primers Vp72 sense (CTG CTC ATG GTA TCA ATC TTA TCG A) and Vp72 antisense (GAT ACC ACA AGA TC[AG] GCC GT) and the probe 5′-(6-carboxyfluorescein [FAM])-CCA CGG GAG GAA TAC CAA CCC AGT G-3′-(6-carboxytetramethylrhodamine [TAMRA]) (44). A standard curve was prepared from a p72 mimic plasmid by making serial dilution ranging from 10^8^ to 10^1^ copies/mL. Results were reported as log_10_ genome copies/mL.

### Antibody responses.

The level of antibody responses against ASFV-p72 in serum was measured using a commercial blocking ELISA (INgezim PPA Compac, Ingenasa) following the manufacturer’s instructions. The percentage of blocking (PB) was calculated using the following formula: [(negative-control OD − sample OD)/(negative-control OD − positive-control OD)] × 100, where OD is optical density. Samples were considered positive if the PB was above the cutoff value of 50% blocking.

### IFN-gamma ELISpot assay.

Peripheral blood mononuclear cells (PBMCs) were collected at 3 different time points, including preimmunization, preboost, and prechallenge. PBMCs were purified from EDTA blood tubes using Histopaque-1083 or -1077 gradient medium. ELISpot plates were coated overnight at 4°C with 4 μg/mL IFN-γ monoclonal antibody (P2F6, Invitrogen, ThermoFisher Scientific) in 0.05 M carbonate-bicarbonate coating buffer. After incubation, plates were washed four times with phosphate-buffered saline (PBS). Cells were plated in duplicate at two different dilutions (8 × 10^5^ and 4 × 10^5^ per well), in RPMI 1640, Glutamax (Gibco) supplemented with 10% fetal bovine serum, 50 μM 2-mercaptoethanol, 100 IU/mL penicillin, and 100 μg/mL streptomycin. The cells were then incubated overnight at 37°C in a final volume of 200 μL with 10^5^ HAD_50_ of Benin 97/1, an equivalent volume of mock inoculum, or 20 μg/mL phytohemagglutinin as a positive control. Cells were lysed by incubation for 5 min in water and then washed with PBS. Following incubations with biotinylated anti-porcine IFN-γ monoclonal antibody (P2C11, Invitrogen ThermoFisher Scientific) and streptavidin conjugated to alkaline phosphatase, AP conjugate substrate kit (Bio-Rad) was used to develop spots. The spot-forming cells were then counted using an ELISpot assay reader system (Immunospot, CTL). The number of spots per well was converted into the number of spots per million cells, and the mean for duplicate wells was plotted using GraphPad Prism 8 software. No cells were collected for pigs belonging to group D.

### Statistical analysis.

Statistical analysis was performed using GraphPad Prism8 software. Two-way analysis of variance (ANOVA) followed by Dunnett’s multiple-comparison test was performed to evaluate the *in vitro* growth differences between the ASFV isolates, while Sidak’s multiple-comparison test was used to evaluate the differences in the levels of IFN-γ-producing cells between groups.
